# Supporting obstetric sonographers following diagnostic error: Evidence-informed interventions to promote well-being, learning and patient safety

**DOI:** 10.1177/1742271X261489050

**Published:** 2026-10-02

**Authors:** Elaine Gardiner, Julie Roberts, Evelyn McElhinney, Thelma Okey-Adibe, Morgyn Kay

**Affiliations:** 1 Glasgow Caledonian University, UK

**Keywords:** obstetric ultrasound, diagnostic error, second victim phenomenon, sonographer well-being, support interventions, patient safety, trauma-informed care, healthcare leadership

## Abstract

**Introduction:**

Diagnostic errors in obstetric ultrasound can cause significant harm to patients and are increasingly recognised to have profound emotional and professional consequences for clinicians, described as the second victim phenomenon. An empirical study of obstetric sonographers identified substantial second victim distress and highlighted gaps in preparation, support and organisational response. This paper translates those findings into practical, evidence-informed interventions for clinical practice.

**Methods:**

An abductive, pragmatic intervention development approach was used, informed by empirical findings and relevant literature on second victim support, trauma-informed care and just culture. A structured mapping process linked identified gaps in knowledge, skills, policy and resources to proposed intervention components.

**Results:**

Three complementary intervention models were developed. Model 1 proposes a supportive learning environment to better prepare sonographers for diagnostic error through education, peer support and structured learning opportunities. Model 2 presents an Ultrasound Manager Second Victim Support Tool to guide consistent and compassionate managerial responses. Model 3 outlines a phase-based early intervention guideline to support timely, trauma-informed responses following diagnostic error, including immediate, follow-up and ongoing support.

**Conclusion:**

These models provide a practical framework to support sonographers and ultrasound managers following diagnostic error. By addressing identified gaps in preparation, managerial response and early support, they aim to promote well-being, learning and patient safety, and support the development of just, learning-orientated cultures. Future research should evaluate these interventions in clinical practice.

## Introduction

Diagnostic errors in obstetric ultrasound remain a recognised source of preventable harm within maternity care, with potentially devastating consequences for mothers, babies and families.^1,2^ While patients are the primary victims, there is increasing recognition that healthcare professionals may also experience significant emotional, psychological and professional consequences. This is commonly described as the second victim phenomenon.^3,4^ Emotional responses may include guilt, shame, fear, intrusive memories and symptoms consistent with burnout and post-traumatic stress, particularly when adequate support is lacking.^5,6^

Obstetric sonographers work in high-risk, high-pressure environments. Their role involves real-time diagnostic decision-making, subjective interpretation, time constraints and significant emotional labour when communicating both reassuring and distressing findings.^7,8^ However, structured organisational responses to diagnostic error in ultrasound practice remain limited. As a result, support mechanisms vary widely across services.^9,10^

A recent empirical study of obstetric sonographers found substantial second victim distress, professional self-doubt and burnout among obstetric sonographers.^11^ It also identified inconsistent managerial support and strong calls for a learning-orientated, just culture. Participants highlighted gaps in education, skills, policy and resources, particularly in psychological first aid, transparent investigations and access to support.

However, understanding lived experience alone is not sufficient. Evidence suggests that improving workforce well-being and patient safety requires translation into practical, structured interventions.^12,13^ Existing second victim support programmes are largely designed for medical or nursing staff, often lack specificity for imaging professionals and do not reflect the unique clinical and emotional context of obstetric ultrasound practice.^14^

This paper develops evidence-informed, practice-focused interventions to support obstetric sonographers and ultrasound managers before and after diagnostic error. These interventions address key stages of the error trajectory, including preparation for practice, managerial response and early post-error support.

## Methods

### Study design

This intervention development study formed part of a wider programme of research investigating the impact of diagnostic error on obstetric sonographers. Findings from a previous empirical study^11^ were used as one of several data sources informing intervention development.

This paper develops evidence-informed intervention models to support obstetric sonographers following diagnostic error. Building on previous empirical findings,^11^ an abductive and pragmatic approach was used. This allowed iterative movement between empirical data, theory and professional guidance to produce contextually relevant and feasible interventions.^15,16^

### Intervention development approach

Intervention development followed principles of translational, implementation-focused research. Empirical findings were used to develop practical solutions addressing identified problems.^13,17^ The aim was not to test effectiveness, but to design conceptually robust, evidence-informed models aligned with second victim, trauma-informed and just culture frameworks.^12,18^

This approach ensured the interventions were theoretically informed while remaining grounded in real-world obstetric ultrasound practice.^13,19^

### Data sources informing intervention design

The intervention models were developed using three complementary sources of evidence.

First, empirical findings from the previous wider study were reviewed to identify gaps in knowledge, skills and organisational processes and support. There was a particular focus on participants’ narratives where distress was either intensified or alleviated by organisational responses, as reflective evidence supports that the quality of the response significantly influences second victim recovery.^5,12^

Second, relevant literature and conceptual frameworks were integrated, including research on the second victim phenomenon, peer support, trauma-informed care and just culture.^12,13,20^ Where ultrasound-specific guidance was limited, wider healthcare literature was used to inform intervention design.^18,21^

Third, practice and policy guidance were consulted to enhance feasibility. This included psychosocial support guidance developed during the COVID-19 pandemic,^22,23^ recommendations from the European Researchers’ Network Working on Second Victims^14^ and workplace mental health guidance from the Chartered Institute of Personnel and Development.^14^ These sources provided strategies for psychological first aid, phased support and organisational implementation that complemented the empirical findings.

To ensure transparency and methodological rigour, a structured mapping process was used to link identified gaps to specific intervention components. First, key findings from the empirical study were grouped into recurring support needs, including preparation for diagnostic error, managerial response, emotional support and organisational learning. Second, these needs were compared against evidence from second victim, trauma-informed care and just culture literature to identify potential intervention mechanisms. Third, intervention components were mapped according to whether they addressed pre-error preparedness, manager response or post-error recovery support. This process enabled explicit linkage between identified needs and proposed intervention elements. For example, limited awareness of the second victim phenomenon and inconsistent managerial responses informed the development of the manager support tool and early intervention guideline.^13,17^ Figure 1 illustrates the intervention development and mapping process used to translate empirical findings and evidence synthesis into targeted intervention models.

**Figure 1.**
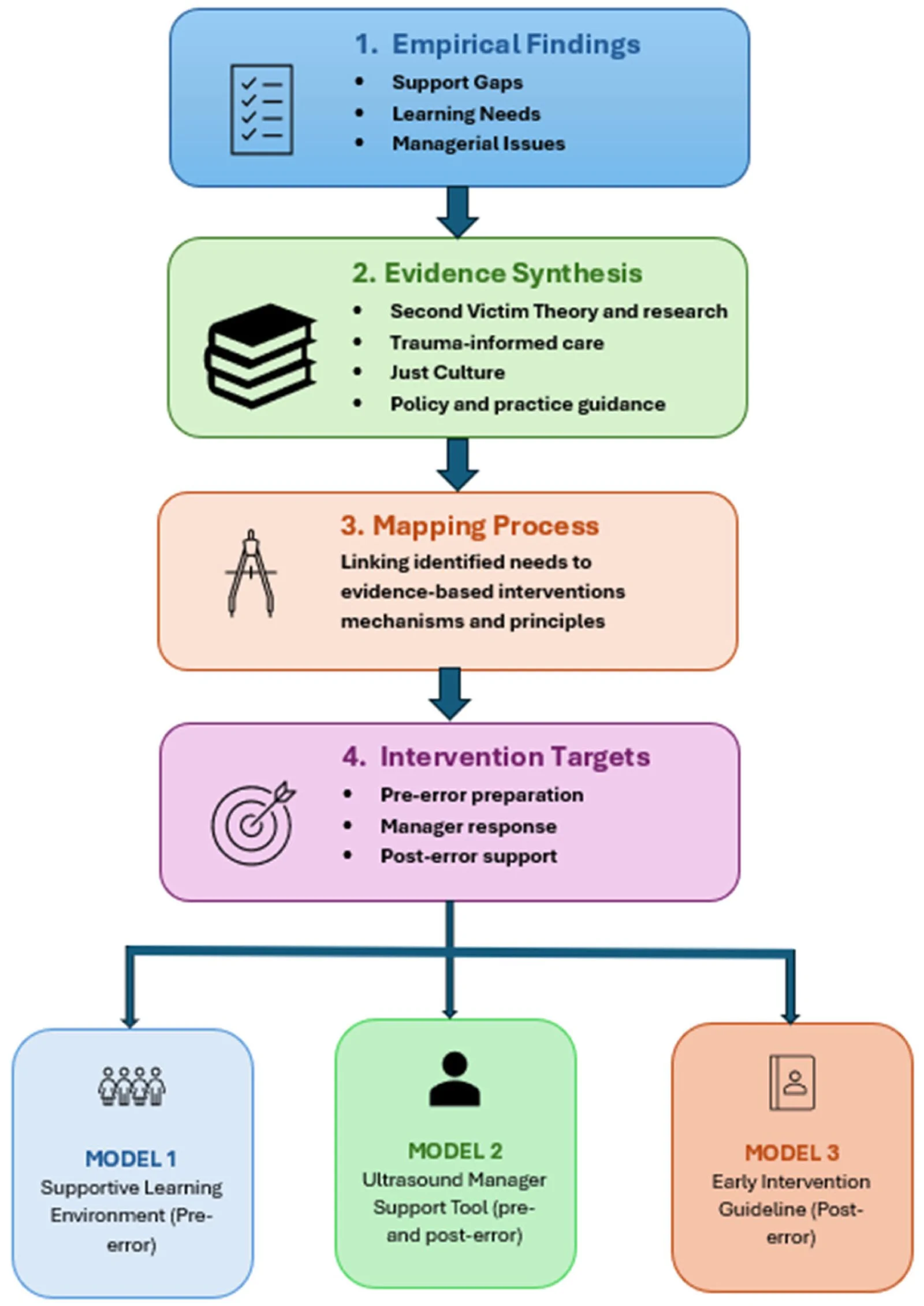
Intervention development and mapping process.

Through iterative synthesis of these sources, three complementary intervention approaches were developed, addressing pre-error preparation, managerial support and early post-error response. Models were refined to ensure clarity, feasibility and alignment with trauma-informed and just culture principles.^15,24^ Visual representations were developed to support accessibility and implementation.

### Ethical considerations

No new data were collected for this phase. Ethical approval for the overall study, including use of empirical findings for intervention development, was granted by an institutional research ethics committee (Code: HLS/PSWAHS/20/220) was obtained from the Glasgow Caledonian University’s School of Health and Life Sciences). All data were anonymised and handled in accordance with the approved procedures.

## Results: Evidence-informed intervention models

Three models were developed: Model 1 (supportive learning environment), Model 2 (manager support tool) and Model 3 (early intervention guidance). The models were derived from empirical findings from a previous study and synthesised with existing second victim and trauma-informed frameworks through the mapping process shown in Figure 1. Collectively, these interventions target key stages influencing second victim outcomes: preparedness before error, the quality of the manager response, and the timing and structure of early support following error.

### Relationship between the intervention models

The three models were intentionally developed as complementary rather than competing interventions. Each addresses a different stage of the diagnostic error trajectory.

Model 1 focused on preparedness before an error occurs. Model 2 focuses on managerial response surrounding an error, and Model 3 focuses on immediate and ongoing support following error recognition.

Although individual elements may be adopted separately according to local need and resources, implementation of all three models is recommended because together they provide a comprehensive support pathway spanning prevention, response and recovery.

To enhance transparency, Table 1 illustrates how key findings from the empirical study were mapped against relevant literature and conceptual framework to inform the development of the three intervention models. This process ensured that proposed intervention components were grounded in both stakeholder experiences and established evidence.

**Table 1.** Mapping empirical findings and literature to intervention models.

Identified gap	Empirical findings	Supporting literature/frameworks	Intervention model
Limited awareness of second victim phenomenon	Participants reported little understanding of the emotional impact of diagnostic error	Second Victim recovery frameworks; prevention focused approaches^11,12^	Model 1
Lack of preparation for diagnostic error	Sonographers described feeling unprepared for the consequences of error	Psychological safety and anticipatory learning literature^12,13^	Model 1
Limited opportunities for shared learning	Participants requested structured opportunities for reflection and learning	Just culture and organisational learning approaches^17,20^	Model 1
Inconsistent managerial responses	Experiences varied considerably depending on individual managers	Leadership and second victim recovery literature	Model 2
Fear of blame during investigations	Participants described punitive or procedural responses	Just culture frameworks^17,20,25^	Model 2
Poor immediate support following error recognition	Distress was exacerbated by insensitive communication and lack of support	Psychological first aid and early intervention guidance^19,21,22^	Model 3
Lack of structured follow-up support	Some participants reported prolonged distress with little ongoing support	Recovery trajectory and phased support models^11,12,19^	Model 3

### Model 1: Establishing a supportive learning environment (pre-error)

The first intervention focuses on the pre-error phase and aims to prepare sonographers for the emotional and professional impact of diagnostic error. It incorporates education, peer support and structured learning opportunities. Findings indicated limited awareness of the second victim phenomenon, gaps in preparation and restricted opportunities for shared learning. These informed the model design.

The model includes six interrelated components: education on diagnostic error, second victim phenomenon, mental health and well-being training, peer support, sonographer-led learning (Sonographer Events and Learning Meetings (SEALM)) and multidisciplinary engagement. Collectively, these aim to normalise fallibility, reduce stigma, enhance psychological safety and promote reflective learning.

By embedding this model within training, professional development and organisational culture, it aims to mitigate the impact of diagnostic error, enhance resilience and support sustainable ultrasound practice (Figure 2). Model 1, along with accompanying guidance, can be accessed in the Supplementary Material (Supplementary File 1).

**Figure 2.**
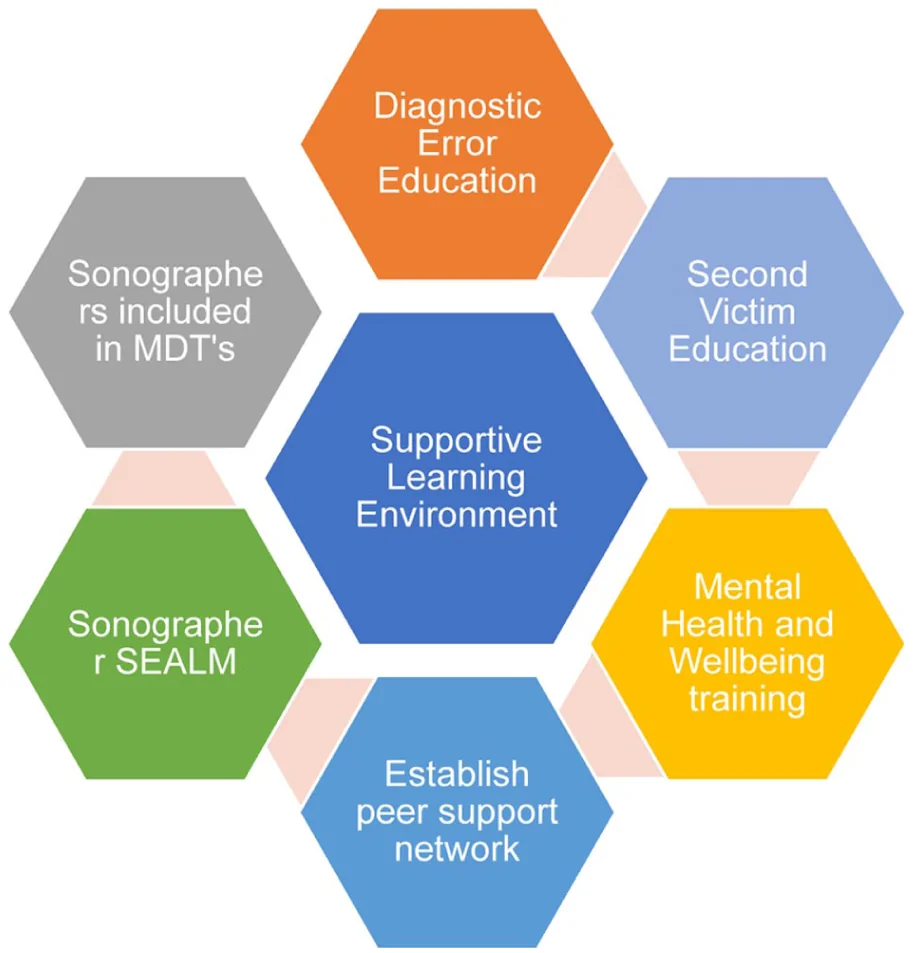
Model 1: establishing a supportive learning environment.

### Model 2: Ultrasound manager support tool (pre- and post-error)

The second intervention recognises the pivotal role ultrasound managers have in shaping the emotional climate and support mechanisms within ultrasound departments. Findings showed that managerial responses were often inconsistent and perceived as procedural rather than supportive.

This model provides a structural tool organised across four domains: *education and training, investigation processes, safe space* and *support*. It guides managers to deliver compassionate, timely and consistent responses while maintaining psychological safety.

By developing managers’ skills in psychological first aid and communication, this model aims to reduce second victim distress and promote empathetic, learning-orientated management practice (Figure 3). Model 2, along with accompanying guidance, can be accessed in the Supplementary Material (Supplementary File 2).

**Figure 3.**
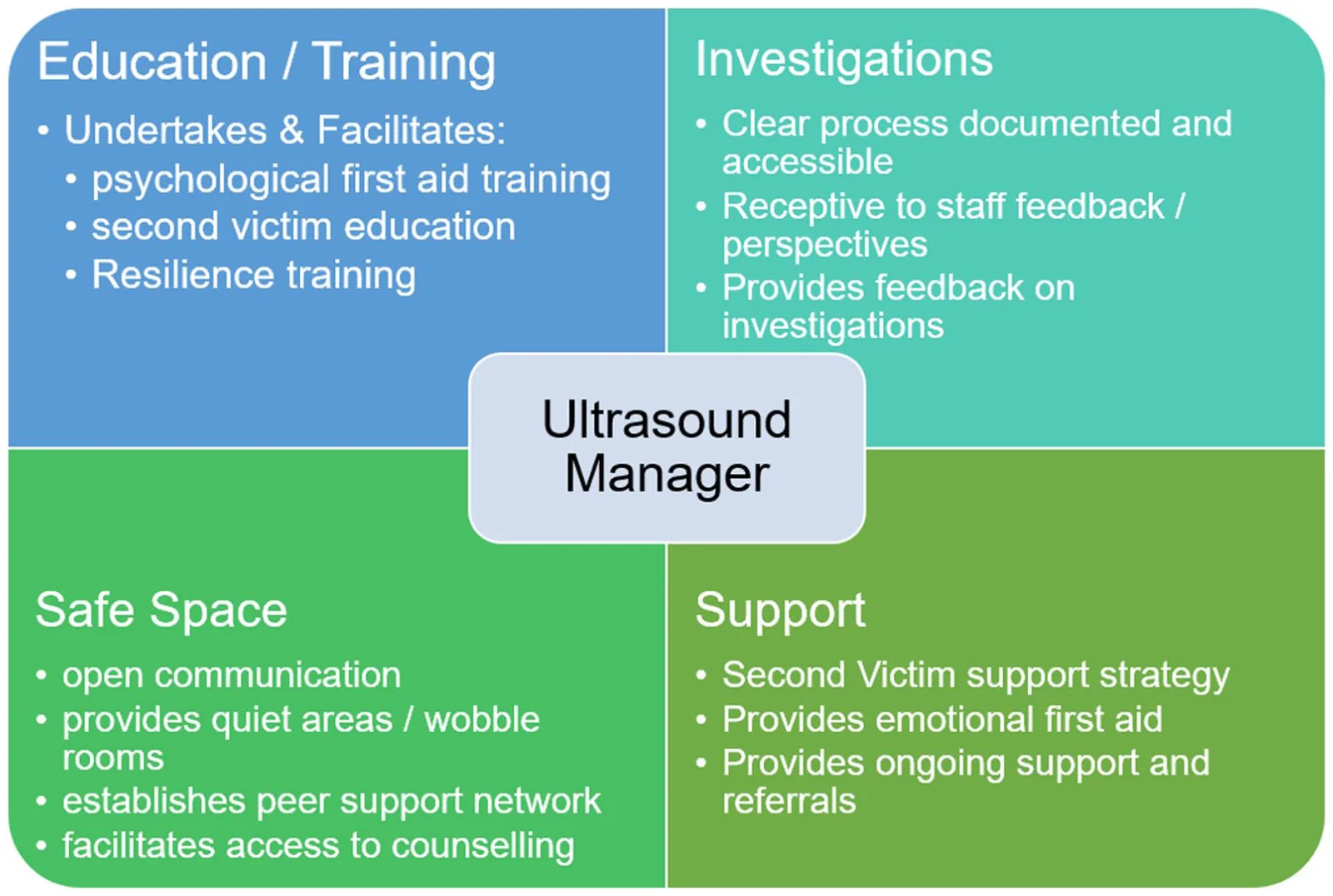
Model 2: ultrasound manager support tool.

### Model 3: Early intervention guidance for ultrasound managers

The third intervention provides phase-based guidance for managers responding to diagnostic error. It addresses inconsistencies in communication, particularly during the immediate aftermath of learning about an error.

The guideline is structured across three phases: Pre-error preparedness, initial response following diagnostic error, and follow-up and ongoing support. It emphasises empathy, psychological first aid, appropriate communication and follow-up care.

Underpinned by trauma-informed and just culture principles, the guideline aims to standardise early supportive responses, reduce psychological harm, and foster trust between sonographers and management. The early intervention guideline can be accessed in the Supplementary Material (Supplementary File 3).

## Discussion

This paper introduces three evidence-informed intervention models developed to support obstetric sonographers following diagnostic error. These interventions address gaps in awareness, skills, organisational processes and support identified in a previous empirical study,^11^ that contribute to second victim distress. These experiences may also be understood through the lens of emotional labour and moral distress, where clinicians experience conflict between professional responsibility and perceived personal failure following diagnostic error.^4,6^ Collectively, they move beyond recognising harm towards practical, trauma-informed strategies that promote well-being, learning and patient safety within obstetric ultrasound services. Rather than functioning as discrete interventions, the three models are intended to operate as an integrated framework, combining preparations, managerial support and early post-error intervention to strengthen individual and organisational responses to diagnostic error.

### Addressing gaps in support following diagnostic error

The empirical findings underpinning these interventions highlight multiple, interconnected gaps in support. Sonographers described limited awareness of the second victim phenomenon, lack of preparation for error, inconsistent managerial responses, restricted opportunities for learning and inadequate access to emotional support. These findings align with wider second victim literature, showing that insufficient organisational support contributes to prolonged distress, burnout and intention to leave the profession.^5,12,13^ Importantly, supporting clinicians following diagnostic error is not only a well-being priority but also a patient safety strategy, as unresolved distress is associated with reduced performance, defensive practice and increased risk of subsequent error.^5,12^

Collectively, the three intervention models were developed to address these gaps across the diagnostic error trajectory, encompassing preparedness before error, managerial response and early post-error support. The models draw on evidence emphasising psychological safety, leadership, trauma-informed care and early intervention as key determinants of second victim recovery.^12–15,17,20^

Model 1 supports a shift from reactive responses towards preventive, learning-orientated cultures through education, shared learning and normalisation of error. Model 2 builds on evidence highlighting that leadership behaviours are central to second victim recovery and seeks to promote consistent, compassionate responses.

Model 3 extends phase-based recovery frameworks by operationalising early, structured responses tailored to the timing and context of diagnostic error in ultrasound practice. Evidence indicates that early responses strongly influence second victim recovery trajectories.^12,20^

Together, the models address support needs at individual, managerial and organisational levels, promoting well-being, learning and patient safety within obstetric ultrasound practice.

### Comparison with existing second victim and trauma-informed frameworks

The three intervention models align closely with established second victim support frameworks. These include Scott et al.’s multi-tiered model and Seys et al.’s emphasis on prevention, early intervention and sustained support.^12,13^ Internationally, second victim programmes such as the Resilience in Stressful Events (RISE) programme and the forYOU programme have demonstrated the value of structured organisational responses, including peer support, early intervention and access to escalating pathways following adverse events.^13,14,20^ The intervention modules proposed in this study align with these principles but extend them through their specific focus on obstetric ultrasound practice, diagnostic error and the role of ultrasound managers. However, many existing frameworks have been developed in acute medicine or nursing contexts and may not fully capture the unique features of obstetric ultrasound practice, such as delayed error recognition and real-time diagnostic responsibility. In addition, these models often focus on general organisational responses without providing role-specific guidance for managers or sonographers. The current models aim to address these limitations by offering contextually tailored, role-specific interventions. Collectively, the interventions operationalise primary, secondary and tertiary levels of support within a context-specific framework.^12,13^

In addition, the models are grounded in trauma-informed care principles. These include recognition of emotional impact, prioritisation of psychological safety, empathetic communication and avoidance of re-traumatisation.^21^ Rather than framing distress as individual weakness, the intervention models acknowledge the normality of emotional responses to error. They also emphasise shared responsibility and organisational learning.

Furthermore, the emphasis on openness, learning and fairness reflects key principles of a Just culture, which promotes system-focused investigation while maintaining accountability and compassion.^18,21,26^ In the empirical study informing this work, sonographers expressed strong dissatisfaction with blame-orientated cultures and secretive practices. In contrast, the proposed models aim to create environments where errors can be discussed constructively without fear, supporting both well-being and patient safety.

### Novel contribution to obstetric ultrasound practice

While second victim interventions are described in broader healthcare contexts, these models are specifically tailored to obstetric ultrasound practice. This represents a shift from descriptive accounts of second victim experiences towards actionable, role-specific intervention design within a previously under-researched professional group.

They account for unique aspects of sonography, including real-time diagnostic decision-making, delayed awareness of error, responsibility for both the mother and her unborn child, and the emotional demands of delivering difficult findings.

A further contribution is the focus on role-specific interventions, addressing both sonographers and ultrasound managers. Previous research often focuses on individual coping or peer support alone. In contrast, this study highlights the importance of leadership, organisational processes and workplace environment in shaping recovery. This reframes support as a system-level responsibility rather than an individual burden.

### Implementation considerations

Although formal evaluation of these interventions lies beyond the scope of this paper, implementation feasibility was considered throughout development. The models are designed to be adaptable to local contexts and scalable across ultrasound services with varying resources. However, successful implementation will depend on organisational commitment, leadership engagement and access to training.

Potential barriers include workforce shortages, time pressures and entrenched blame cultures. These challenges have been widely reported in ultrasound services.^9,10^ Engagement with professional bodies, educators and service leaders may support implementation and sustainability. Future research should focus on piloting the models, evaluating acceptability and impact, and refining implementation strategies with key stakeholders.

From an implementation perspective, the proposed models align with principles of behaviour change and organisational learning,^13,17^ recognising that sustainable practice change requires not only knowledge provision but reinforcement through leadership, culture and systems. Embedding these interventions within routine training, supervision and governance structures may support normalisation of supportive practices and reduce reliance on individual initiative.

## Strengths and limitations

A key strength of this work is that the interventions are grounded in empirical data from obstetric sonographers’ lived experiences. Although not formally co-produced, the interventions were grounded in clinicians’ narratives, ensuring alignment with the priorities and experiences of obstetric sonographers. This enhances both relevance and credibility. The integration of these findings with established frameworks and policy guidance further strengthens the conceptual basis of the models.

However, the interventions have not yet been formally tested in clinical settings. Their effectiveness in reducing distress or improving retention therefore remains to be established. In addition, while developed within a UK and Ireland context, adaptation may be required for use in other healthcare systems.

## Conclusions

This paper presents three complementary, evidence-informed intervention models designed to support obstetric sonographers and ultrasound managers before and after diagnostic error. The models address gaps in preparation, managerial response and early support, and are aligned with trauma-informed and just culture principles. By translating empirical findings into practical frameworks, this work contributes to the development of compassionate, learning-orientated ultrasound services that prioritise staff well-being, patient safety and workforce sustainability.

## Supplemental Material

sj-pdf-1-ult-10.1177_1742271X261489050 – Supplemental material for Supporting obstetric sonographers following diagnostic error: Evidence-informed interventions to promote well-being, learning and patient safety Supplemental material, sj-pdf-1-ult-10.1177_1742271X261489050 for Supporting obstetric sonographers following diagnostic error: Evidence-informed interventions to promote well-being, learning and patient safety by Elaine Gardiner, Julie Roberts, Evelyn McElhinney, Thelma Okey-Adibe and Morgyn Kay in Ultrasound
