# Supplementary material for: Supporting obstetric sonographers following diagnostic error: Evidence-informed interventions to promote well-being, learning and patient safety
Source: Ultrasound. 2026 Oct 2:1742271X261489050. Online ahead of print. doi: 10.1177/1742271X261489050 (PMC13646191; doi:10.1177/1742271X261489050)
Supplement: Supplementary file 1 [file sj-pdf-1-ult-10.1177_1742271X261489050.pdf]

Early Intervention Guidance for Ultrasound Managers Supporting Second Victim Sonographers following Diagnostic Error.

The following guidance is not an exhaustive list of recommendations but is intended to inform ultrasound managers of the processes that may be helpful, or unhelpful, in supporting Sonographers emotional well-being following a diagnostic error.

Background

Errors in antenatal obstetric care can have devastating consequences for both the mother and child, leading to severe health complications, disability and even death (1). Misdiagnosis and/or delay in diagnosis are significant contributors to preventable harm in obstetric practice and this includes errors in ultrasound diagnosis (2). Due to the subjective nature of ultrasound scanning and interpretation, it is more prone to errors than other imaging modalities (3), potentially leading to misdiagnosis or mismanagement of the pregnancy.

Sources of error are multi-factorial and may be due to systematic factors such as the equipment used, lighting conditions, length of shift and pace of workload or cognitive factors such as errors in clinical decision making and cognitive biases (4). Maternal obesity is also reported to be a contributing factor (5) and some conditions may not present until late in pregnancy, after the 20-week anomaly scan (6).

When a diagnostic error occurs both the patient and healthcare professional are affected. The patient is the first victim, however the healthcare professional is often referred to as the second victim if they have been profoundly affected or traumatised following the error (7 and 8). Sonographers across the UK and Ireland who have made a diagnostic error have reported experiencing second victim responses including self-doubt, guilt, fear, flashbacks and poor concentration; symptoms associated with burnout and post-traumatic stress disorder (PTSD). They also report that the level of support that they received was poor and their work relationships and job satisfaction were negatively affected. They also expressed a desire to leave their Sonography post.

The level and quality of support that second victims receive and the environment in which they work can significantly influence the level of distress that they experience following an error (9). Support for the sonographer should begin in their own unit or service with psychological first aid provided by their manager and colleagues.

This guideline aims to support ultrasound managers develop a supportive learning environment and support their Sonographer's emotional well-being prior to, and after a diagnostic error.

Fostering a Supportive Learning Environment – Pre-Error - Do's

✓	Provide training on diagnostic error and second victim phenomena and 'normal' responses
✓	Foster a culture of openness, normalising learning from incidents and errors. Facilitate Sonographers attending local MDT's / morbidity and mortality meetings
✓	Provide mental health and well-being training to help staff develop individualised resilience and coping strategies.
✓	Facilitate Sonographer-led Events and Learning Meetings (SEALM), suggest 3-weekly, lunch-time, learning event highlighting 'good spots' (unusual pathology or findings), departmental audit, interesting cases / feedback on study days and diagnostic errors to 'normalise' errors
✓	Do facilitate team cohesion and try to foster strong supportive links between team members and managers. Connections and peer support are important factors in developing peer support and resilience.
✓	Provide opportunities for staff to talk about difficult encounters or significant experiences at the end of their shift or significant points, staff should not feel mandated to talk about their thoughts or feelings
✓	Develop / provide clear documentation on the local departmental and organisational investigation processes, demonstrating that investigations are fair, transparent and learning focused
✓	Establish links / fast track referrals to the organisations counselling / well-being service
✓	Identify suitable staff members who could be trained and act as peer supporters
✓	Manager and Sonographers identified as 'peer supporters' to complete psychological first aid (PSA) training
✓	Provide a 'safe' space, where there is open, confidential communication and a quiet area / 'wobble' room

Initial Meeting Discussing the Error - Do's

✓	Arrange a suitable time (not at start of a scan list) and quiet location to meet with Sonographer

✓	Arrange temporary work adjustments to ensure Sonographer is free of workload and not returning to scan (i.e. break, non-patient facing task, home)
✓	Be present physically and emotionally – ensure no distractions or interruptions
✓	Reassure the Sonographer that all discussions are confidential and that they are not alone in their experience
✓	Ensure that the information provided about the particular case and type of error is clear and accurate.
✓	Use ‘we’ statements to foster a collaborative approach to finding solutions and sense of shared responsibility
✓	Give the Sonographer the opportunity to process the information, be prepared for silences and be patient
✓	Ask how they are feeling – ask open, non-judgemental questions
✓	Actively listen and show you are receiving their answer with empathy and understanding
✓	Validate their feelings - acknowledge how stressful it is learning about an error and how it can trigger strong emotions
✓	Explain the potential emotional and physical responses the Sonographer may experience and how to recognise them.
✓	Show understanding of what they are going through, share a personal experience (if appropriate).
✓	Discuss self-care activities and resources and potential peer support from colleagues
✓	Provide information of support offered within the organisation, such as employee assistance programme / counselling
✓	Offer clear information on investigation process (if appropriate)
✓	Discuss temporary work adjustments such as time away
✓	Ask if they would like any information about the error shared with colleagues and how, as this can be very supportive for some people.
✓	Arrange a follow-up meeting within one week

Don’ts

X	Don’t hold meetings in scan room
X	Don’t appoint blame
X	Don’t ask questions such as:<br><br>You’re clearly struggling, what’s up?<br><br>Everyone else is in the same boat and they’re okay, why aren’t you?
X	Avoid making assumptions about how they will respond,
X	Don’t fill silences immediately, give the Sonographer time to think, use silence to encourage further sharing
X	Don’t rush to offer formal psychological interventions / counselling too soon as this may intervene with their natural coping mechanisms and be detrimental

Follow-up Meetings and Ongoing Care / Recovery:

✓	Continue to have meetings in a suitable quiet environment
✓	Continue to provide reassurance and actively listen
✓	Continue to actively monitor Sonographers well-being
✓	Continue to provide emotional first aid and support (focus on the Sonographer emotions (not the error)
✓	Be flexible in supporting needs and respond to feedback on what is, and is not helpful for them
✓	If concerned that second victim responses are persisting (after 4 weeks), guide them to additional / formal support resources
✓	Explore sharing / developing learning resources based on incident

References / Bibliography:

1 NHS Resolution (2025) *Annual report and accounts 2024/25 (HC 1065).* London: NHS Resolution. Available at: https://assets.publishing.service.gov.uk/media/687a2dae312ee8a5f0806b6f/nhs-resolution-annual-report-and-accounts-2024-to-2025-hc1065-web-accessible.pdf

2 Krenitsky, N.M., Perez-Urbano, I. and Goffman, D. (2024) 'Diagnostic errors in obstetric morbidity and mortality: Methods for and challenges in seeking diagnostic excellence', *Journal of Clinical Medicine,* 13(14), article 4245. Available at: https://doi.org/10.3390/jcm13144245

3 Di Serafino M, Iacobellis F, Schillirò ML, D'auria D, Verde F, Grimaldi D, Dell'Aversano Orabona G, Caruso M, Sabatino V, Rinaldo C, Guerriero P, Cantisani V, Vallone G, Romano L. (2022) Common and Uncommon Errors in Emergency Ultrasound. *Diagnostics* (Basel). 4;12(3):631. doi: 10.3390/diagnostics12030631.

4 Sotiriadis, A. and Odibo, A.O. (2019) 'Systematic error and cognitive bias in obstetric ultrasound', *The Official Journal of the International Society of Ultrasound in Obstetrics and Gynecology*, 53(4), pp. 431-435. Available at: https://doi.org/10.1002/uog.20232

5 Brunner, K., Linder, T., Klaritsch, P., Tura, A., Windsperger, K. and Göbl, C. (2025) 'The impact of overweight and obesity on pregnancy: A narrative review of physiological consequences, risks and challenges in prenatal care, and early intervention strategies', *Current Diabetes Reports*, 25, article 30. Available at: https://doi.org/10.1007/s11892-025-01585-3

6 Alfirevic, Z. (2005) 'DISQ 3: Failure to diagnose a fetal anomaly on a routine ultrasound scan at 20 weeks', *Ultrasound in Obstetrics & Gynaecology,* 26(7), pp. 797-798. Available at: https://doi.org/10.1002/uog.2631

7 Wu, A.W., Shapiro, J., Harrison, R., Scott, S.D., Connors, C., Kenney, L. and Vanhaecht, K. (2020) 'The impact of adverse events on clinicians: What's in a name?', *Journal of Patient Safety*, 16(1), pp. 65-72. Available at: https://doi.org/10.1097/pts.0000000000000256

8 Nydoo, P., Pillay, B.J., Naicker, T. and Moodley, J. (2019) 'The second victim phenomenon in health care: A literature review', *Scandinavian Journal of Public Health*, 48(6), pp. 629-637. Available at: https://doi.org/10.1177/1403494819855506

9 Kaur (et al 2019) Kaur, P.A., Levinson, A.T., Monteiro, J.F.G. and Carino, G.P. (2019) 'The impact of errors on healthcare professionals in the critical care setting', *Journal of Critical Care*, 52, pp. 16-21. Available at: https://doi.org/10.1016/j.jcrc.2019.03.001
